# Comparative Study of Thermal Accumulation and Adaptive Responses of *Forsythia × intermedia* Zabel and *Kerria japonica* (L.) DC. ‘Pleniflora’ Under Conditions of Climatic Fluctuations

**DOI:** 10.3390/plants15172682

**Published:** 2026-08-31

**Authors:** Djurdja Petrov, Dragan Vujičić, Nevenka Galečić, Dejan Skočajić, Jelena Čukanović, Radenka Kolarov, Mirjana Ocokoljić

**Affiliations:** 1 Faculty of Forestry, University of Belgrade, Kneza Viseslava 1, 11030 Belgrade, Serbia; dragan.vujicic@sfb.bg.ac.rs (D.V.); nevenka.galecic@sfb.bg.ac.rs (N.G.); dejan.skocajic@sfb.bg.ac.rs (D.S.); mirjana.ocokoljic@sfb.bg.ac.rs (M.O.); 2 Faculty of Agriculture, University of Novi Sad, Trg Dositeja Obradovića 8, 21000 Novi Sad, Serbia; jelena.cukanovic@polj.uns.ac.rs (J.Č.); radenka.kolarov@polj.uns.ac.rs (R.K.)

**Keywords:** Border Forsythia, Double Japanese Rose, plants in extreme environments, biodiversity, acclimatization, flowering phenological patterns, phenological synchronization

## Abstract

The study establishes a locally specific basis that may serve as an indicator for the selection of climate-adaptive taxa in urban landscape design through a comparative evaluation of the effects of thermal anomalies on the flowering phenodynamics of *Forsythia × intermedia* and *Kerria japonica* ‘Pleniflora’ in Belgrade (2007–2026). By integrating empirical phenological parameters into a long-term climatological retrospective, the study bridges a methodological gap and distinguishes anthropogenic influences from regional climatic fluctuations. Non-parametric analyses identified a stable long-term trend, while the absence of statistically significant macro-scale effects confirms the predominant role of natural climatic variability. Nevertheless, microclimatic modifications induce distinct, taxon-specific phenological responses. Non-linear regression models identified two divergent adaptive mechanisms during warmer years: a phenological compression model in *F. × intermedia* (shortening of the flowering cycle accompanied by an annual increase in growing degree days (GDDs) of 0.71%) and a non-linear expansion model in *K. japonica* ‘Pleniflora’ (extension of the flowering period by more than one month, with the end of flowering delayed by 8.4 days per decade). Circular statistical analyses confirmed a high degree of temporal synchronisation and genetically determined phenological rhythmicity in both taxa, while revealing asynchrony in the terminal phenophases that was independent of growing degree day (GDD) accumulation. The synchronous shift towards warmer thermal classes positions both taxa as bioindicators of microclimatic change, although *K. japonica* ‘Pleniflora’ exhibited greater adaptive capacity. These findings highlight the importance of applying non-linear models in phenological forecasting and a contextualised taxonomic and ecological basis for the planning, introduction, and management of green spaces, supporting the sustainability of shrub taxa within the analysed urban core.

## 1. Introduction

Urban ecosystems represent distinct, dynamic, and anthropogenically modified environments in which the intensification of the urban heat island (UHI) effect induces primary abiotic transformations of local thermal regimes [1]. These changes directly affect urban vegetation, most notably through alterations in spring phenological patterns. In the urban environment of Belgrade, chronological shifts and earlier onset of spring phenophases have been empirically documented in economically and ecologically important woody taxa, showing a direct correlation with atmospheric warming trends [2,3,4]. Understanding these changes is essential for quantifying the vulnerability of urban greenery and designing effective mitigation strategies [5,6].

In the context of urban ecology, the urban environment functions as an in situ laboratory [7], providing an opportunity to monitor the adaptive and ecophysiological responses of plants that determine the vitality and sustainability of green infrastructure [8]. In woody taxa from temperate continental climates, the onset and timing of flowering are regulated by complex temperature-mediated processes. Elevated winter and early-spring temperatures primarily affect the release of plants from endo- and ecodormancy, as well as the process of vernalisation [9]. The rate at which chilling requirements are fulfilled during winter, followed by successive heat accumulation in early spring, directly determines the chronology, dynamics, and duration of flowering, placing temperature responses at the forefront of contemporary research [10,11].

Particularly sensitive model systems for monitoring these responses are hysteranthous taxa, which flower in early spring before the development of foliage [12]. Because their reproductive organs are directly exposed to ambient conditions without the thermal insulation provided by vegetative structures, they function as sensitive bioindicators of microclimatic changes, responding directly to temperature fluctuations [13]. Although extreme weather events, such as late frosts, may induce delays in phenophase development [14,15], altered phenological patterns serve as primary biological indicators of the magnitude of local thermal changes [16].

Across the broader Balkan Peninsula, and particularly within the urban core of Belgrade, winter and spring seasons are characterised by pronounced climatic non-stationarity, resulting in fluctuations in local thermal regimes during the colder periods of the year [17,18]. Therefore, monitoring and statistical classification of seasonal temperatures against the World Meteorological Organization’s standard climatological reference periods are essential for informing adaptation strategies for urban green spaces [19,20]. Given that early-spring flowering plants respond rapidly to warming and that temperature dynamics influence the duration of flowering [21], this study uses a continuous 20-year observational dataset (2007–2026) to analyse the effects of temperature changes on *Forsythia* × *intermedia* (Oleaceae) and *Kerria japonica* ‘Pleniflora’ (Rosaceae). While taxa of the genus Forsythia Vahl, as typically hysteranthous plants, exhibit specific response patterns to spring anomalies [22], the long-term persistence of introduced cultivars such as *K. japonica* ‘Pleniflora’ depends primarily on their phenological plasticity and capacity to buffer microclimatic extremes [23].

The primary objective of this study is to establish a robust empirical basis for the selection of climate-adaptive ornamental shrub taxa for urban landscape design. The study focuses on the comparative evaluation of the effects of identified thermal anomalies on the timing of phenophases (day of year—DOY) and the precise quantification of heat requirements using accumulated growing degree days (GDDs). Through the application of nonlinear models, the study seeks to identify divergent adaptation mechanisms, reflected in the compression or expansion of the flowering period depending on the rate of heat-unit accumulation. In this way, the research addresses a methodological gap in the existing literature on the phenological plasticity of urban vegetation in Southeast Europe and provides a scientifically grounded basis for the sustainable management of urban green spaces.

The primary objective of this research is to establish an accurate empirical basis that enables urban planners, landscape architects, and ecologists to select climate-adaptive taxa from the group of hysteranthous species suitable for integration into specific urban micro-locations. Within this context, the study focuses on the comparative evaluation of the effects of identified thermal anomalies and extreme seasonal (winter and spring) fluctuations on the chronological shifts in flowering phenological patterns of *F. × intermedia* (Border Forsythia) and *K. japonica* ‘Pleniflora’ (Double Japanese Rose), including a precise quantification of their thermal requirements. In addition, the study aims to determine the influence of climatic fluctuations on the flowering phenodynamics of urban ornamental shrub taxa. In this way, the research identifies and addresses gaps in the existing literature, contributing to a better understanding of the adaptive responses of urban vegetation to pressures caused by changing climatic conditions, while also establishing a stronger empirical foundation for future research.

## 2. Results

### 2.1. Categorisation of Winter and Spring Seasons: Identification of Anomalies

**Figure 1:**
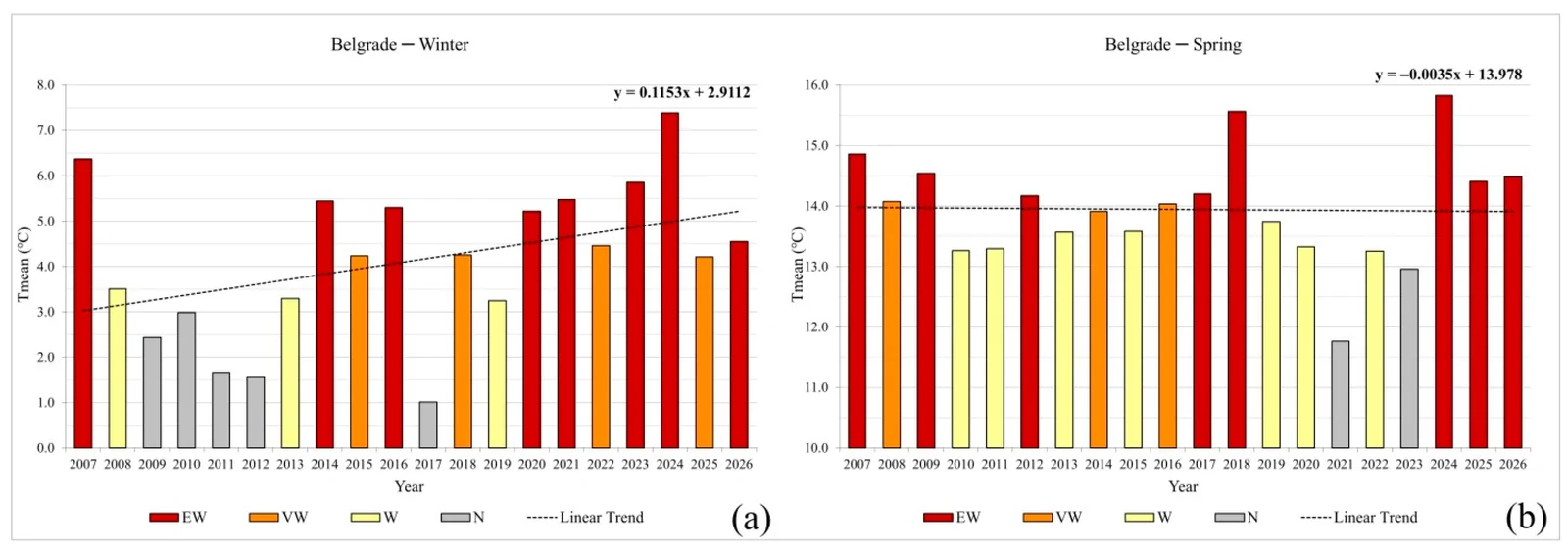
Classification of mean winter (**a**) and spring (**b**) air temperatures for the study years (2007–2026), based on the percentile method, relative to the reference period 1961–1990. Legend: EW (extremely warm), VW (very warm), W (warm), and N (normal).

Analysis of the multi-year time series (2007–2026) in Belgrade indicates a predominance of positive thermal anomalies during the winter and spring months relative to the climatological normal (Figure 1a,b). Over the 20-year observation period, traditional winters and springs classified as “within the normal range” were reduced to a historical minimum, while categories ranging from “warm” to “extremely warm” became a persistent feature, with no seasons classified as “cold”. Pronounced anomalous warm extremes were recorded repeatedly during the last two decades, culminating in the final years of the observation period, including 2026. Despite the pronounced visual fluctuations and high mean seasonal temperatures, the non-parametric statistical analysis (Table 1) does not confirm the presence of a statistically significant long-term trend in the analysed 20-year time series.

The Mann–Kendall (MK) test values for the winter seasons and their corresponding *p*-values (*p* > 0.05) indicate interannual variations that are consistent with natural variability. This is further supported by the Sen’s slope (0.136), which is close to zero and indicates the stability of the positive-anomaly regime. Similarly, the spring trends show no statistically significant change (*p* > 0.05), with a minimal Sen’s slope of −0.007. The slight visual decline in spring temperatures shown in the graph is attributable to within-decadal variability and temperature extremes in individual years (such as 2021 and 2023), rather than to long-term climatic cooling.

This redistribution of climatic classes in Belgrade (2007–2026) relative to the reference period (1961–1990) provides empirical evidence of intensifying local microclimatic modifications under the influence of the urban heat island (UHI) effect. The substantial reduction in seasons within the normal range and the clear predominance of warm extremes indicate a shift in the local climate regime relative to the standards established at the end of the 20th century.

### 2.2. Flowering Patterns of Forsythia × intermedia and Kerria japonica ‘Pleniflora’

Analysis of the 20-year phenological observations (2007–2026) reveals taxon-specific differences in the adaptive potential and phenodynamic responses of the studied taxa to microclimatic fluctuations within the urban ecosystem.

**Figure 2:**
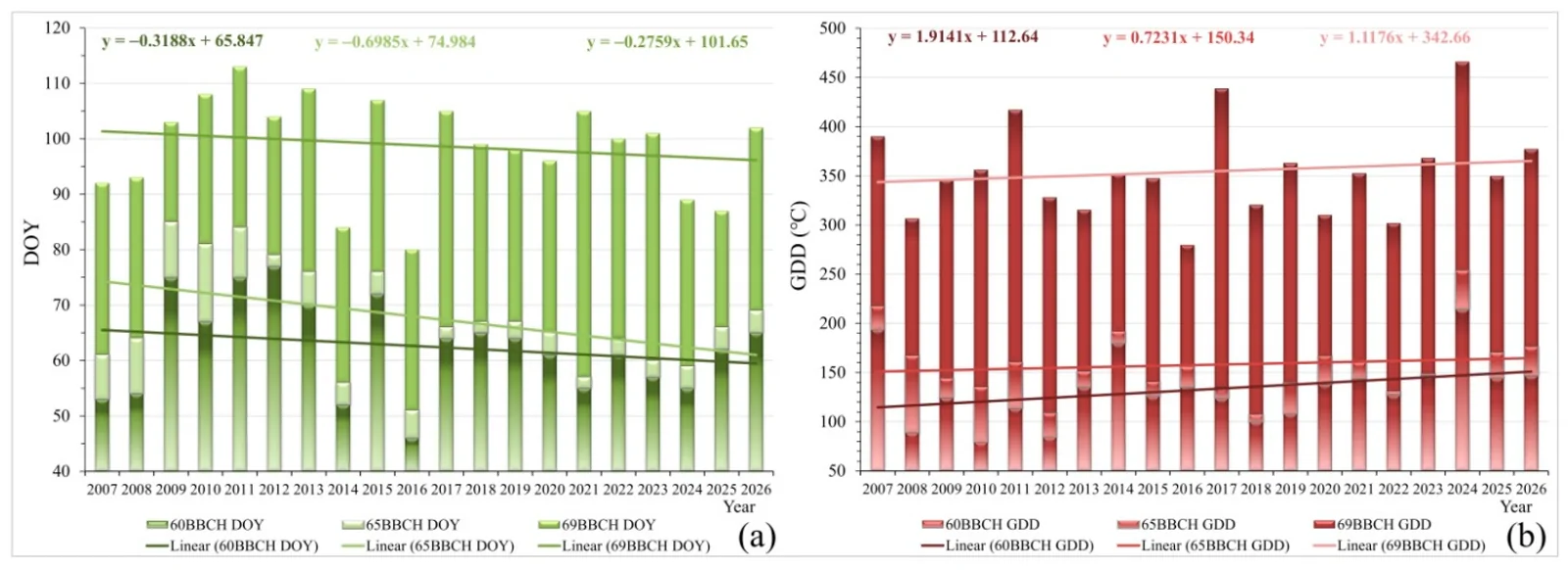
Chronological representation of the phenological patterns of primary flowering of Border Forsythia, with corresponding linear trends, in Belgrade for the period 2007–2026, expressed as: (**a**) DOY and (**b**) GDD (°C), for the successive stages of the onset (BBCH 60), full flowering (BBCH 65), and end of flowering (BBCH 69). Legend: BBCH (Biologische Bundesanstalt, Bundessortenamt und CHemische Industriescale), DOY (day of year), GDD (growing degree days).

*F. × intermedia* exhibits a synchronised, rapid flowering strategy before leaf emergence, serving as an indicator of early-spring atmospheric warming. Over the 20-year observation period, all phenophases are represented by BBCH—Biologische Bundesanstalt, Bundessortenamt und CHemische Industriescale (BBCH 60, 65 and 69) showed visually apparent declining trends in the calendar days of occurrence (DOY), indicating an advance in flowering, accompanied by a corresponding upward trend in GDD—the accumulated sums of effective temperatures (Figure 2).

Application of the Mann–Kendall and Sen’s slope tests showed that the observed linear trends were generally not statistically significant, indicating a strong influence of natural interannual variability (Table S1). The exception was a highly significant positive trend in the accumulation of thermal units (GDD) required for flowering initiation (BBCH 60). The Sen’s slope value (2.306) indicates a consistent annual increase in the thermal sum required to overcome ecodormancy. This phenomenon suggests that the more rapid increase in spring temperatures may enable the earlier attainment of internal thermal thresholds, or that climatic fluctuations may be modifying the thermal requirements of the taxon itself. The Spearman correlation coefficient (Table S2) confirms a strong positive linear relationship between the calendar timing of flowering onset and full flowering, as well as moderately strong negative correlations between earlier phenophase occurrence (DOY) and increased heat accumulation requirements (GDD).

**Figure 3:**
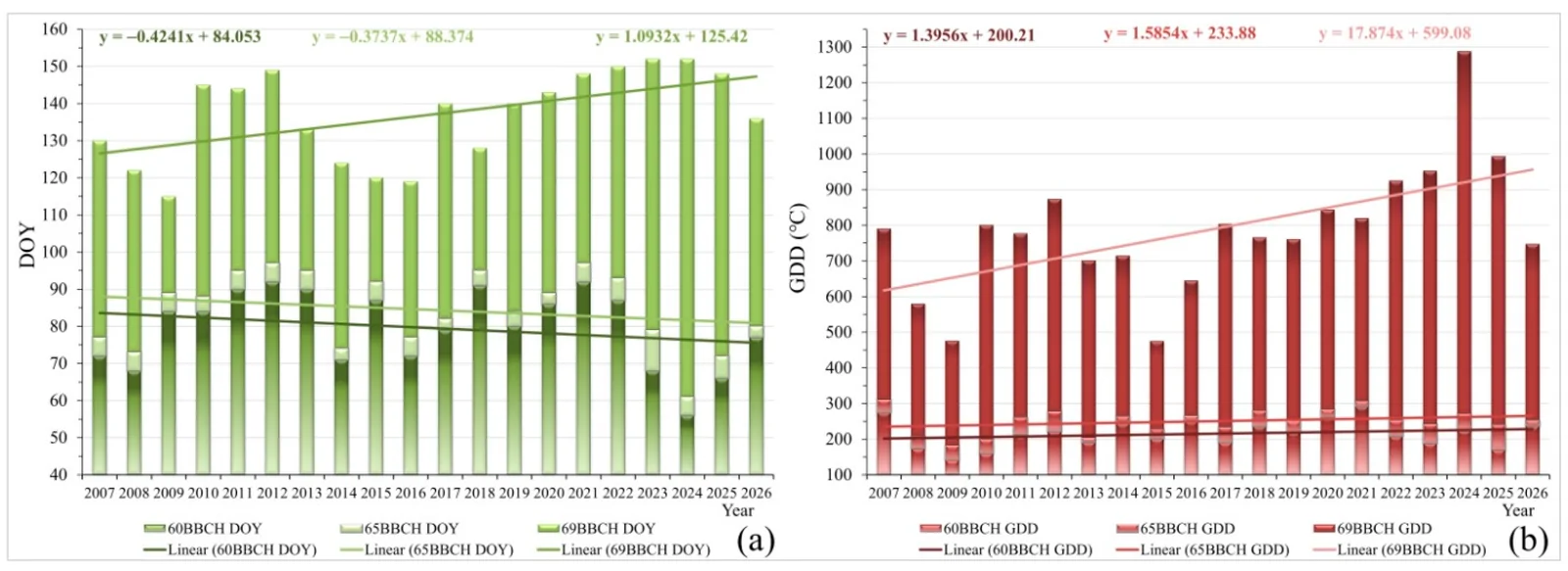
Phenological patterns and linear trends of primary flowering of the cultivar *Kerria japonica* ‘Pleniflora’ in Belgrade (2007–2026). The BBCH 60, 65, and 69 phases are graphically presented in relation to: (**a**) day of the year (DOY) and (**b**) accumulated temperature (GDD). Legend: BBCH (Biologische Bundesanstalt, Bundessortenamt und CHemische Industriescale).

In contrast to the previous taxon, the cultivar *K. japonica* ‘Pleniflora’ exhibited an asynchronous and extended flowering phase that chronologically overlaps with the development of foliage (Figure 3). Monitoring results indicate an earlier onset of both flowering initiation and full flowering (declining DOY trend), but also a specific upward trend in the end of flowering (BBCH 69), resulting in a progressive extension of the overall flowering duration under the influence of the urban heat island (UHI) effect. Non-parametric analysis (Table S1) confirmed statistically significant trends (*p* < 0.05) exclusively for the final flowering phase (BBCH 69). The Sen’s slope for calendar time (1.243) confirms a linear trend towards delayed flowering cessation, amounting to approximately 24 days over the entire study period. At the same time, the high Sen’s slope for thermal sums (17.299) indicates that the cultivar reaches the end of flowering under considerably warmer conditions, as a result of accelerated spring warming and altered physiological efficiency under elevated temperatures. The Spearman correlation matrix (Table S2) identified strong, highly selective, positive relationships between the calendar timing of flowering onset and full flowering, as well as between DOY and GDD values during the final flowering phase (BBCH 69).

Quantitative analysis of the phenological responses clearly distinguishes two divergent adaptive strategies. While *F. × intermedia* responds through phenodynamic compression of the flowering cycle, *K. japonica* ‘Pleniflora’, owing to the successive opening of flower buds, exhibits non-linear phenological expansion. Its high phenological plasticity enables *K. japonica* ‘Pleniflora’ to more effectively buffer the effects of late-spring frosts and heat stress; nevertheless, both introduced taxa demonstrate their value as reliable bioindicators of anthropogenically modified microclimates in urban environments.

### 2.3. Comparative Climatological and Phenological Analysis

**Figure 4:**
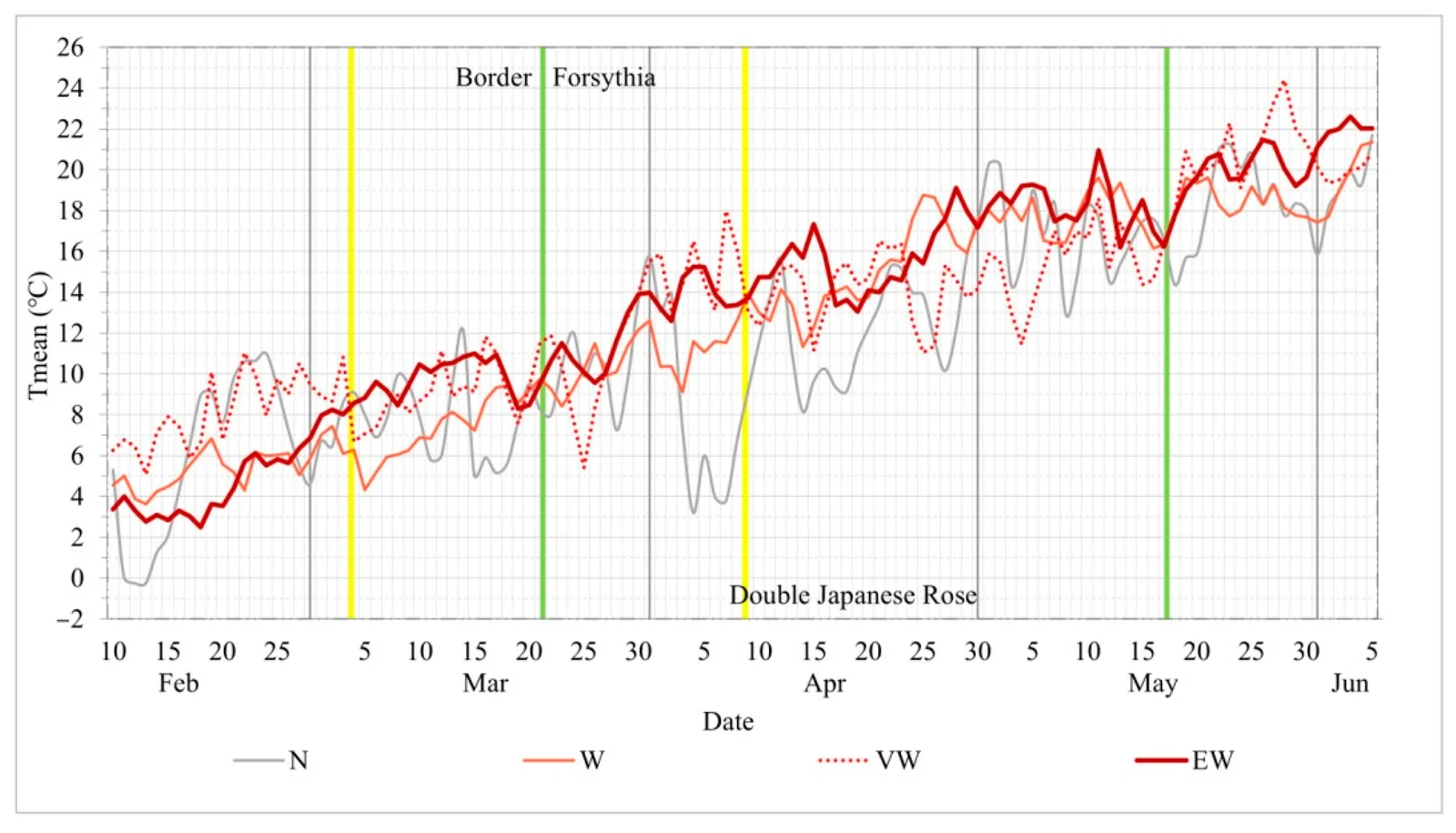
Distribution of mean daily air temperatures (Tmean) by year, classified into the categories extremely warm (EW), very warm (VW), warm (W), and within the normal range (N) for the period 2007–2026, relative to the reference climatological period 1961–1990, with the projected mean twenty-year flowering periods of Border Forsythia (yellow lines) and Double Japanese Rose (green lines) indicated.

Analysis of mean daily air temperatures during the primary flowering period (15 February–1 June) across different year classes (EW—extremely warm, VW—very warm, W—warm and N—normal range) indicates that phenodynamic patterns are strongly influenced by the altered thermal regime (Figure 4).

The projected flowering periods of both analysed taxa chronologically coincide with a high frequency of days characterised by positive thermal anomalies. The early-spring flowering of *F. × intermedia*, as well as the late-spring flowering period of *K. japonica* ‘Pleniflora’, consistently coincide with classes of warm and extremely warm days.

Comparative synthesis confirms that the flowering phenophases of both taxa occur under thermal conditions that consistently and substantially exceed the historical norms of the baseline period (1961–1990). This persistent shift towards higher temperature classes (W, VW, EW) demonstrates the exposure of urban vegetation to elevated thermal stress and confirms a synchronous adaptive response of both taxa to microclimatic changes in Belgrade.

#### 2.3.1. Phenological Shifts and Changes in Thermal Requirements

Non-linear regression models (Table 2) for “very warm” (VW and EW) and “warm” (W) year classes reveal taxon-specific phenodynamic responses to temperature fluctuations.

The non-linear regression equations for *F. × intermedia* indicate that early flowering reaches a biological limit under extremely warm conditions. The minimal changes in the calendar date (DOY 60) suggest that, despite further increases in temperature, the taxon cannot initiate flowering substantially earlier than late February. In contrast, the transition to full flowering and flowering cessation occurs progressively earlier and at an accelerated, non-linear rate, with high spring temperatures acting as a catalyst for phenophase progression.

Under “warm” years, the full-flowering stage of this taxon responds most rapidly to warming, advancing more quickly than flowering onset itself. This asymmetric exponential shift results in a progressive shortening of the overall flowering period (cycle compression) by an average of 6.2 days. The accelerated progression through phenophases is accompanied by a distinct divergence in thermal requirements: while the threshold of accumulated growing degree days (GDDs) required for flowering initiation shows a consistent exponential increase, the GDD requirements for the full-flowering and flowering-cessation stages decline, creating a pronounced risk of late-spring frost damage.

The cultivar *K. japonica* ‘Pleniflora’ exhibits a markedly different strategy under “very warm” and “warm” years. While flowering onset and full flowering shift exponentially towards earlier dates in spring as a result of more rapid late-winter warming, flowering cessation (BBCH 69) exhibits a stable non-linear increase. Under the influence of climatic fluctuations, flowering cessation is consistently delayed, extending the flowering period by more than 20 days (approximately 8.4 days per decade), resulting in an extension of the overall flowering duration by more than one month (cycle expansion).

The extended phenological window is accompanied by a consistent non-linear increase in the accumulation of thermal units (GDD) across all three observed phenophases, without a shortening of the overall flowering cycle. While the early stages retain stable and moderate thermal requirements, the terminal stage requires intensive and accelerated heat accumulation under elevated spring temperatures. The successive opening of flower buds under considerably warmer late-spring conditions provides mathematical evidence of the cultivar’s high phenological plasticity and superior capacity to buffer microclimatic anomalies associated with the urban heat island effect.

#### 2.3.2. Circular Statistical Indicators of Phenological Seasonality and Flowering Synchrony

Application of the Rayleigh test of uniformity (Table 3) confirms a high temporal concentration, stable seasonal rhythmicity, and pronounced synchronisation of all analysed calendar phenophases (DOY) in both taxa over the 20-year period. High mean resultant vector length (r) values indicate minimal interannual calendar variability. In *F. × intermedia*, a rapid phenological pattern was verified, with a swift transition from flowering onset to full flowering within six days, while the slight decrease in vector concentration at the end of flowering (BBCH 69) in both taxa reflects a marginally greater dependence of the terminal stages on prevailing microclimatic conditions.

Comparative circular analysis of heat accumulation (GDD) reveals a divergent pattern of temporal synchronisation. At the onset (BBCH 60) and during full flowering (BBCH 65), both taxa show a high concentration of observations around the mean angle, demonstrating that optimal heat sums are a primary and stable trigger of the early stages of reproductive development.

However, a key biological difference was identified during the terminal flowering stage (BBCH 69). While *F. × intermedia* retains statistically significant uniformity driven by the dynamics of GDD accumulation, the Rayleigh test for *K. japonica* ‘Pleniflora’ yields low and statistically non-significant concentration vector values. This finding provides evidence that the timing of flowering cessation in the Double Japanese Rose is highly dispersed across years and is primarily controlled by other factors or extreme microclimatic anomalies, rather than by heat accumulation alone.

**Figure 5:**
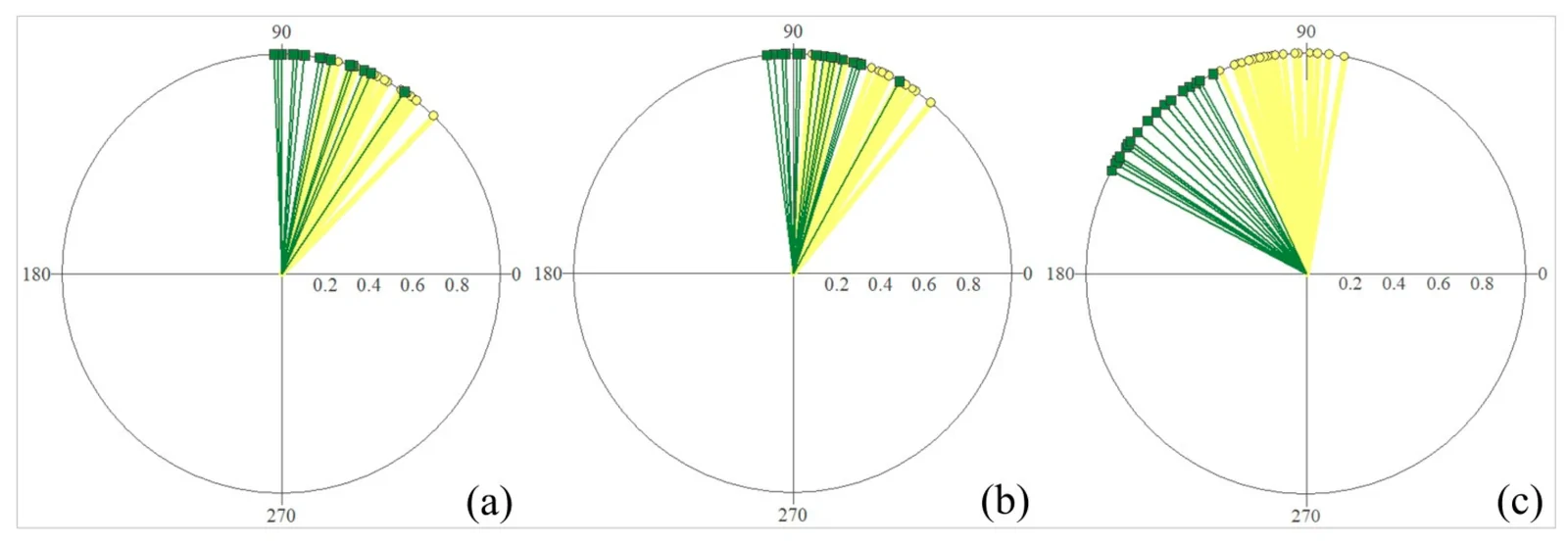
Seasonal dynamics of flowering phenological phases of *Forsythia × intermedia* (yellow lines) and *Kerria japonica* ‘Pleniflora’ (green lines) according to the Watson–Williams test during the period 2007–2026 in Belgrade, expressed as DOY (day of year): (**a**) onset of flowering (BBCH 60), (**b**) full flowering (BBCH 65), and (**c**) end of flowering (BBCH 69).

Seasonal and thermal differentiation was further verified using the Watson–Williams tests (Table 4, Figure 5). Comparative analysis confirmed highly statistically significant differences between the taxa across all phenophases for both indicators (DOY and GDD). The greatest asynchrony in calendar time (DOY) was identified at the end of flowering, indicating taxon-specific responses to late-spring environmental conditions. In contrast, with respect to thermal requirements (GDD), maximum divergence was observed during full flowering, whereas the values at the end of flowering converged, indicating convergence towards similar final thermal thresholds.

The circular correlation results (Table 3) confirm a strong and direct calendar-based association (DOY) during the initial phenophases, indicating that external seasonal factors synchronously regulate the onset of the growing season in both taxa during the same period of the year. In contrast, the correlation with growing degree days (GDDs) during the terminal flowering stage is statistically negligible and effectively absent, demonstrating that the mechanisms governing flowering cessation do not follow the same thermal response in the two taxa.

From an applied perspective, the observed continuity and stability of the early phenophases provide a scientific basis for the combined integration of *F. × intermedia* and *K. japonica* ‘Pleniflora’ in the design of mixed urban green barriers, ensuring high aesthetic and functional sustainability under pronounced anthropogenic pressure.

## 3. Discussion

### 3.1. Interpretation of Highly Fluctuating Time Series and the Local Context

The discrepancy between the visual perception of upward/downward trends in the graphs and the absence of macro-statistical significance (*p* > 0.05) over the 20-year period (2007–2026) represents a fundamental methodological phenomenon characteristic of highly fluctuating ecological time series. The lack of statistical significance in the Mann–Kendall test, despite the apparent predominance of warm extremes, indicates that the observed fluctuations may still be largely governed by the internal stochastic dynamics of the regional climate system (natural variability), rather than by a persistent, linearly directed external signal [24]. Pronounced interannual variability and the occurrence of extreme warm events at the ends of the observation period can often generate visual artefacts and apparent trends. Under such conditions, robust non-parametric tests can help distinguish long-term climatological variability from short-term, high-frequency fluctuations, thereby reducing the risk of statistical errors and misinterpretation of anthropogenic influence [25].

On the other hand, anchoring the two-decade dataset (2007–2026) to the standard historical reference period (1961–1990), as recommended by the World Meteorological Organization [19], provides a clear chronological perspective that was lacking in previous local studies [26,27]. This methodological approach provides strong empirical evidence of the accelerating intensification of local climatic modifications in the Belgrade area. The observed temperature departure from the historical normal is fully consistent with broader regional trends across the Balkan Peninsula [28] and global projections of intensifying temperature extremes in Europe [29]. The statistically verified reduction in seasons classified as “within the normal range” suggests that the local climate system is becoming less stationary. Conditions that were defined as thermal extremes at the end of the 20th century are becoming part of the prevailing climatological reality in the first quarter of the 21st century, as also supported by comparable studies of urban ecosystems in Central and Eastern Europe [30].

The ecological implications of this regime shift in climate are directly manifested in the destabilisation and desynchronisation of plants’ internal biophysiological rhythms. The atypical pattern of winter seasons, characterised by the absence of prolonged periods of frost and a reduction in snow cover [31], contributes to a shortening of the endodormancy period in urban woody vegetation. The synergistic effect of overall regional warming and the urban heat island (UHI) effect in the core of Belgrade [32] acts as a multiplier of early-spring thermal stress, promoting premature accumulation of effective temperatures. Compression of the spring season and abrupt transitions towards summer-like thermal conditions shorten the optimal temporal window for the stable ontogenetic development of plant reproductive organs, making them particularly vulnerable to subsequent cold spells and late-spring frosts.

### 3.2. Thermal Anomalies and Atypical Trends

The non-linear manifestation of temperature extremes and the occurrence of atypical thermal trends within the observed climate classes can be explained by the complex dynamics of synoptic-scale and atmospheric circulation at the macro- and mesoscales. It has been demonstrated that years characterised by exceptionally high annual mean temperatures may generate more intense and abrupt short-term incursions of Arctic air masses than more stable warm years [33]. Conversely, isolated temperature maxima recorded during seasons climatologically classified as “within the normal range” are primarily attributable to local intra-seasonal effects, such as a sudden reduction in cloud cover and high levels of direct solar radiation during specific months [34]. These microclimatic anomalies create specific thermal pressures within the urban fabric, where anthropogenic structures absorb and re-emit thermal energy, modifying the baseline thermal requirements of vegetation independently of the regional climatological mean.

The ecophysiological implications of thermal anomalies depend on the taxon-specific spring phenological window of plants. Early-flowering and hysteranthous taxa, such as *Forsythia × intermedia*, are particularly sensitive to late-winter and early-spring heatwaves, as elevated temperatures act as a catalyst for the premature attainment of the required threshold of accumulated growing degree days (GDDs) [35]. This premature phenological shift brings reproductive organs into the vulnerable flowering stage considerably earlier than historically typical, thereby increasing the risk of frost-induced damage caused by subsequent spring frosts [36].

In contrast, late-flowering taxa and cultivars with extended flowering periods, such as *Kerria japonica* ‘Pleniflora’, are exposed to chronic heat stress during the transition towards summer, which can disrupt the homeostasis of reproductive organs. High temperatures and excessive heat accumulation during this phase reduce viability, accelerate the desiccation of petals, and shorten the overall duration of flowering [37].

The synchronous yet mechanistically divergent shifts in both taxa beyond the historical baseline period (1961–1990) confirm their role as sensitive bioindicators of microclimatic transformations in urban environments [38]. By incorporating data through the most recent year, 2026, this study provides a novel and empirically robust basis for the re-evaluation and modelling of ornamental woody vegetation resilience in the context of current climate scenarios [39].

#### Non-Linear Modelling of Phenological Responses and Ecophysiological Thresholds of Woody Taxa

Identification of non-linear phenological thresholds in *F.* × *intermedia* during anomalously warm years provides insight into the biological limits of early-spring phenological anticipation. The observed cessation of the advance in flowering onset towards the winter period, even under conditions of continued atmospheric warming, suggests that the taxon has reached its ecophysiological limit [40]. This phenomenon can be explained by evolutionarily determined compensatory mechanisms and the interplay of external signals: although accelerated spring thermal forcing shortens the time required to accumulate the threshold GDD values, internal regulatory mechanisms (primarily the fulfilment of chilling requirements and photoperiodic control) act as a brake, preventing premature initiation of flowering during unfavourable winter months [41,42].

On the other hand, the asymmetry in the rate of phenological shifts observed in *F. × intermedia* provides a new perspective on classical linear phenological paradigms [36,43]. Whereas previous studies have largely treated phenophase shifts as linear processes, thereby masking intra-seasonal divergence [44], the non-linear models applied in this study demonstrate that the full-flowering stage responds most strongly to increasing temperatures, acting as an ecological catalyst that rapidly accelerates the transition between ontogenetic stages. The ecophysiological consequence of this acceleration is a pronounced compression of the overall flowering cycle due to accelerated flower senescence [45], which is partly consistent with the findings reported by Smith et al. [46]. Increased thermal flux in the urban environment disrupts the metabolic homeostasis of reproductive organs, shortens the window for successful pollination, and induces premature petal senescence [35]. This compression may result in trophic and phenological mismatches between plants and the local entomofauna, seriously compromising the stability and reproductive functioning of urban ecosystems [47,48].

The non-linear expansion model detected in the *K. japonica* ‘Pleniflora’ cultivar during warm years represents a departure from the conventional ecophysiological patterns described in the existing literature, which predominantly suggests that global warming universally shortens flowering duration in ornamental shrubs [49,50]. The pronounced extension of flowering can be attributed to the cultivar’s specific anatomical, morphological, and ecophysiological plasticity, which may confer a higher thermal threshold and more efficient thermoregulatory mechanisms, similar to the heat-tolerance mechanisms described by Wang et al. [51] in related taxa within the Rosaceae family. Elevated thermal conditions and microclimatic anomalies in the urban core during late spring may stimulate prolonged apical meristem activity and induce the successive, sustained activation of secondary flower buds along the shoots, thereby extending flowering into the warmer period of the year [52].

The findings support a methodological recommendation to move beyond the assumption of fixed or linear GDD thresholds under climatic extremes. The detection of asymmetric rates among phenophases [53] demonstrates that predictions of the impacts of climate change on urban plants and associated ecosystem services (such as resource availability for pollinators) should evaluate each phenophase separately, using flexible mathematical models. The identification of contrasting strategies between the early-spring-flowering hybrid and the cultivar with an extended flowering period provides a novel empirical basis for the selection and long-term planning of climate-resilient and stable urban green infrastructure.

### 3.3. Seasonal Patterns of Flowering and the Effect of Thermal Regime on Phenological Divergence

The incorporation of circular statistics and the Rayleigh test of uniformity into the analysis of the multi-year time series (2007–2026) provides an innovative mathematical framework that eliminates the “boundary effect” of the calendar year by treating phenophases as continuous cyclical phenomena [54]. The exceptionally high temporal concentration and stability of early-spring phenophases in both taxa demonstrate the existence of phenological homeostasis governed by endogenous evolutionary mechanisms, challenging traditional assumptions of linear and erratic shifts in flowering under the influence of the urban heat island effect [36,55]. Calendar synchronisation and minimal interannual variability in the initial phases indicate that stable genetic thresholds of sensitivity effectively buffer external microclimatic noise within the urban core [56,57]. Even in the sterile cultivar *K. japonica* ‘Pleniflora’, the absence of selective pressure for attracting pollinators through seed production did not compromise the stability and predictability of the phenodynamic response, confirming that early reproductive stages are primarily controlled by fixed endogenous programmes and temperature accumulation [58].

The divergence verified by the Watson–Williams tests provides a new ecological perspective, demonstrating that the degree of phenological divergence between the two taxa varies throughout the spring season. The identified pattern, in which the minimum interspecific distance occurs at full flowering, whereas the terminal stage exhibits the greatest asynchrony, challenges the prevailing scientific focus on flowering onset as the primary bioindicator of climate change [59]. High plasticity and asynchrony during the terminal flowering stage confirm that later spring conditions and associated microclimatic factors may act as more critical differential regulators of flowering duration than the factors responsible for the initiation of the growing season [40]. Early phenophases follow a stable ecological and genetic pattern, whereas flowering cessation and petal senescence are more susceptible to intra-seasonal fluctuations, soil moisture deficits, and intensified anthropogenic pressures within the urban environment [50].

In contrast to calendar time, the dynamics of thermal requirements (GDD) exhibit an inverse non-linear pattern, characterised by maximum divergence during full flowering and pronounced convergence of thermal sums at the end of flowering. The detection of this pattern challenges the traditional assumption of a constant linear relationship between GDD accumulation and phenophase progression throughout the entire flowering cycle [60]. The convergence of thermal requirements towards the end of flowering suggests the existence of an upper thermal threshold or an endogenous “heat saturation” mechanism that uniformly accelerates the senescence of floral structures in both taxa exposed to high temperatures during late spring [61]. The extremely weak, and effectively negligible, correlation at the end of flowering demonstrates the distinction between calendar-based and thermal predictors, confirming that the mechanisms governing flowering cessation in these two taxa do not follow an identical thermal response [62]. From an urban landscape design perspective, the stability of calendar-based patterns provides a scientific basis for the combined use of *F. × intermedia* and *K. japonica* ‘Pleniflora’ in creating structurally and aesthetically sustainable green barriers with a high capacity to adapt to climatic fluctuations.

The 20-year dataset represents a major statistical strength of the study, overcoming the limitations of short-term studies, which often overlook the microevolutionary and adaptive responses of introduced taxa to the urban climate and specific microclimatic conditions [63].

## 4. Materials and Methods

### 4.1. Study Area

**Figure 6:**
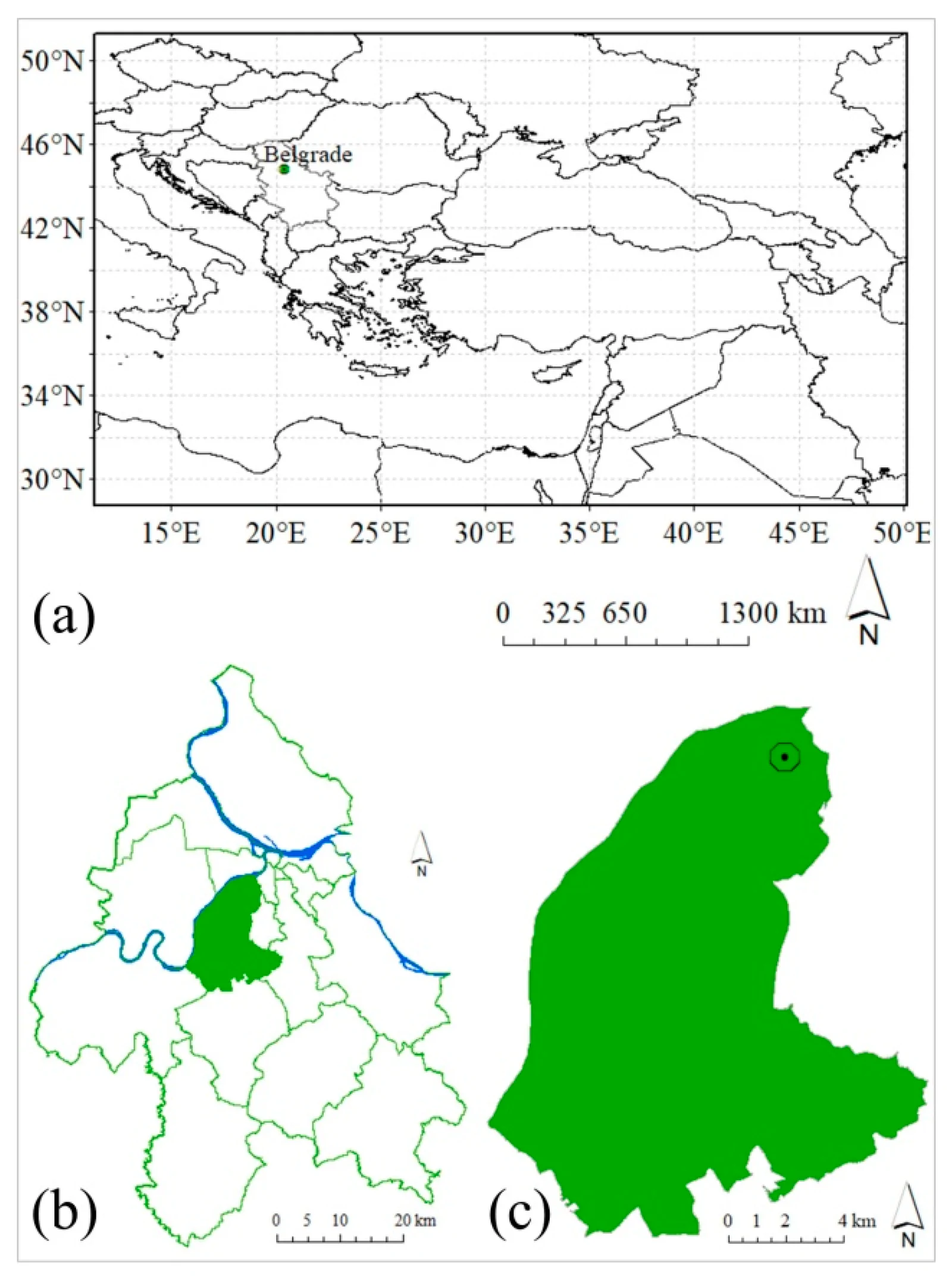
Geographical representation of the study area: (**a**) the geospatial location of the City of Belgrade within Europe; (**b**) the administrative division of the City of Belgrade, highlighting the territory of the Municipality of Čukarica; and (**c**) the territorial extent of the Municipality of Čukarica with the spatial localisation of the investigated area.

The study area is located in Banovo brdo, a morphologically and functionally defined urban sub-centre of Belgrade (Serbia), situated approximately 5.5 km southwest of the city’s historical and administrative core. In terms of planning and morphological organisation, as well as land-use designation, the macro-location is defined by the General Urban Plan of Belgrade [64] as an area within the general urban centre. At a finer spatial scale, the micro-location is situated in front of a building at the very beginning of the Banovo brdo central park (Figure 6), directly adjacent to Požeška Street, and classified as “other types of green areas adjacent to a commercial facility”. Banovo brdo represents a highly urbanised, autonomous secondary centre within the municipality of Čukarica, which functions as a self-sustaining system within the spatial-functional matrix of Belgrade. The primary spatial and economic axis of this sub-centre is Požeška Street, which, according to official planning documentation [65], functions as a linear urban zone of high density. It is characterised by a continuous street frontage formed by multi-storey residential blocks, with predominantly commercial and service activities located on the ground floors. From a transport and structural perspective, Požeška Street represents a major urban corridor connecting the central city areas with southwestern suburban and peripheral settlements. The study area is located at the interface between an intensively used public green space (the central park) and a high-frequency commercial facility, representing a synthesis of microclimatic, ecological, and spatial-social contexts, which makes it a representative site for this research.

**Figure 7:**
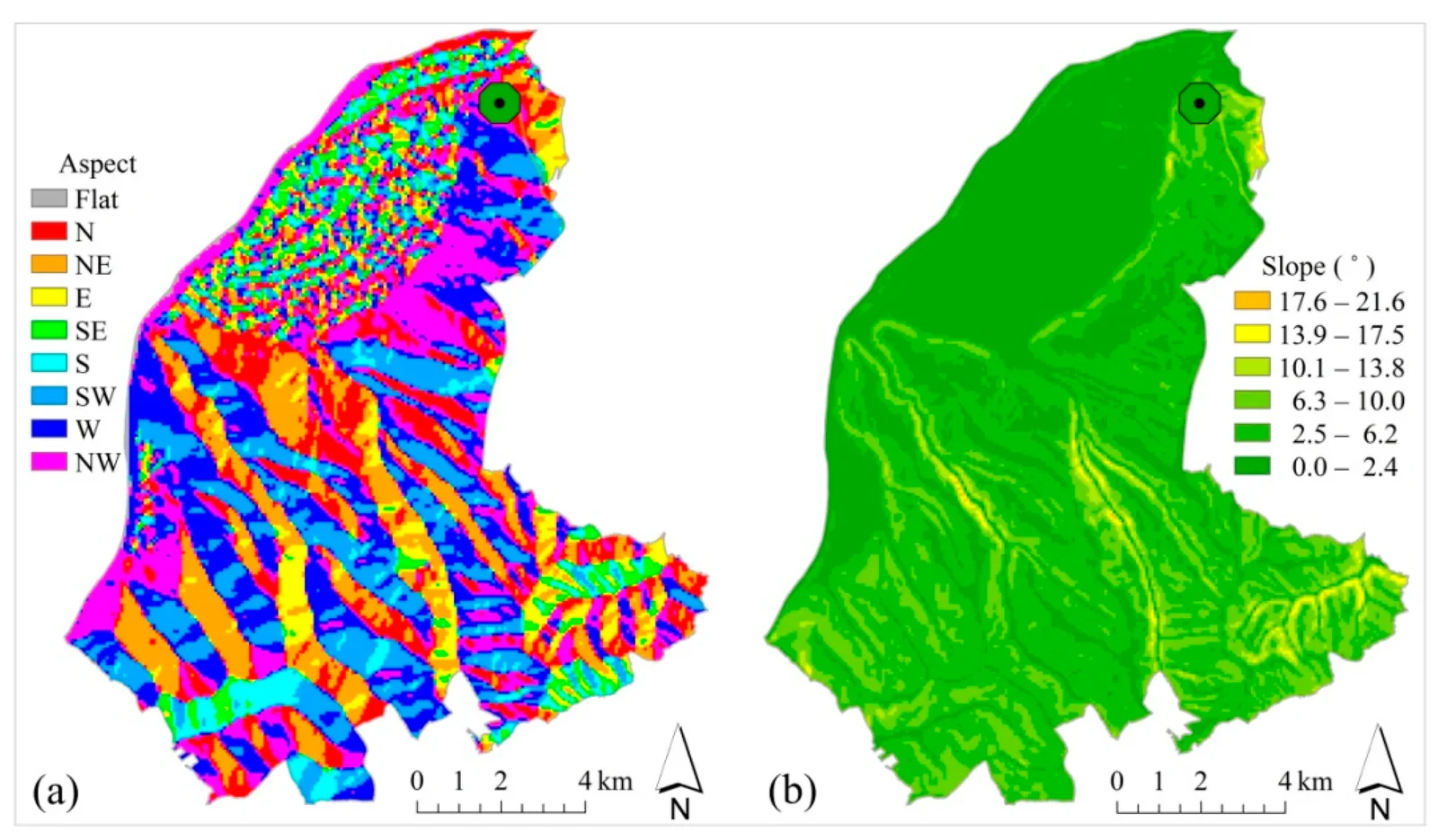
Cartographic representation of terrain aspect (**a**) and slope inclination (**b**) within the territory of the Municipality of Čukarica, with the study area indicated.

During field investigations conducted in 2006, a mixed linear planting structure with a grouped planting arrangement was recorded at the site, in which *Forsythia × intermedia* and *Kerria japonica* ‘Pleniflora’ were selected. The planting micro-location is defined by georeferenced central coordinates of 44°46′51.01″ N and 20°25′02.50″ E, with an average elevation of 122 m a.s.l. The soil substrate on which the linear planting structure is established was classified according to Škorić et al. [66] as anthropogenic chernozem (a leached subtype developed on loess deposits), corresponding to Luvic Chernozems according to the international soil classification system [67]. The orographic characteristics of the terrain (Figure 7) indicate a north-western aspect and a slope inclination of 3.1°, defining the investigated area morphologically as a gently sloping terrain.

#### Plant Material

The study included two widely distributed ornamental taxa: *F. × intermedia* Zabel and *K. japonica* (L.) DC. ‘Pleniflora’. The population groups under observation were located within a landscaped green area in front of a commercial building in the study area. According to information provided by the developer and property owner, the planting material was sourced from local commercial nurseries and planted in autumn 2004 as standard three-year-old trained nursery plants. At the beginning of the field observations and identification of the population groups (2006), the plants were five years old and had reached full functional and ornamental maturity.

Given the specific characteristics of the site, the area was subject to intensive maintenance and care throughout the entire twenty-year period (2007–2026). The maintenance practices included regular irrigation via an automatic drip irrigation system during the dry summer months, as well as the periodic application of compound mineral fertilisers in early spring, thereby ensuring consistent and uniform agrotechnical conditions throughout the two decades.

Pruning practices were carried out in accordance with the biology of both taxa, annually and immediately after the complete end of the flowering phenophase, involving thinning and the removal of spent shoots. For *F. × intermedia*, pruning was carried out in May, whereas for the cultivar *K. japonica* ‘Pleniflora’, it was performed at the end of June. As a result of regular annual pruning, the morphological parameters varied seasonally but remained consistently within the standard ranges characteristic of these taxa under temperate continental climatic conditions: the height of *F. × intermedia* was maintained within the range of 1.8–2.2 m, while that of *K. japonica* ‘Pleniflora’ ranged from 1.5–1.8 m, with a proportional canopy diameter [68,69].

Throughout the entire monitoring period (2007–2026), no phytopathological infections or entomological infestations of significant intensity were recorded in the observed plants that could have affected their natural phenological cycle. All observed individuals maintained the highest vitality rating throughout the study period (Class 0—no signs of decline), according to the modified Roloff vitality scale [70], as well as the highest level of overall ornamental quality.

### 4.2. Meteorological and Phenological Data

The official meteorological data used in this study were obtained from the database of the Republic Hydrometeorological Service of Serbia (RHMS) [71]. The World Meteorological Organization [72] baseline climatological normal period from 1961 to 1990 was applied as the reference climate period, defined as a fixed reference for long-term climate change assessments. This reference period does not overlap with the years of phenological observations, thereby ensuring the methodological independence of the comparative analysis. For this historical thirty-year dataset, climatological standard normals and percentiles for air temperature were calculated at the seasonal level. The analysis of temperature variability over the twenty-year period (2007–2026) was conducted using data from the Main Meteorological Station (MMS) Belgrade (geographical coordinates: 44°47′54.44″ N and 20°27′53.35″ E; elevation: 132 m). To achieve a precise categorisation of winter and spring seasons, the percentile method was applied. As a non-parametric statistical technique, this method represents an objective analytical tool. The advantage of the selected approach lies in its ability to define exact thresholds of extremeness without assuming a normal distribution of data frequencies. This methodological framework is essential for the reliable detection and quantification of temperature anomalies and is fully aligned with the current standards and recommendations of the World Meteorological Organization [73]. According to the official RHMS methodology, the classification of mean daily, monthly, and seasonal air temperatures based on percentiles is defined through seven categories: EW—Extremely Warm (>98th percentile), VW—Very Warm (>90–≤98th percentile), W—Warm (>75–≤90th percentile), N—Normal (≥25–≤75th percentile), C—Cold (≥10–<25th percentile), VC—Very Cold (≥2–<10th percentile), and EC—Extremely Cold (<2nd percentile).

The phenological data analysed in this study were obtained through continuous in situ monitoring of the flowering phenophases of both taxa within the investigated area over a twenty-year period (2007–2026). The field observation protocol involved site visits every second day, while following the registration of the onset of flowering, monitoring was conducted daily until the complete termination of this phenophase. The total dataset comprised 8300 phenological observations. The identification of key flowering stages was performed using the extended BBCH scale [74], with the following stages monitored: the beginning of flowering (BBCH 60: ≥10% of flowers open), full flowering (BBCH 65: ≥50% of flowers open), and the end of flowering (BBCH 69: ≥80% of flowers faded). For statistical analysis, the calendar dates of the recorded phenophases were converted into day of the year (DOY) values, where 1 January corresponds to DOY 1 and 2 January to DOY 2, continuing sequentially throughout the year. Since the analysis of flowering phenological patterns included three representative population groups for each investigated taxon, the DOY values for individual flowering stages were presented and analysed as mean values calculated at the overall population level for each respective taxon. To identify the key phenological stages, the concept of accumulated active temperatures, i.e., growing degree days (GDDs), was applied in accordance with the methodology and improved formulas proposed by Lalić et al. [75]. The calculation of GDD values was performed using daily maximum (Tmax) and minimum (Tmin) air temperatures. A base temperature (temperature threshold) of Tt = 5 °C was adopted, following the official recommendation of the World Meteorological Organization [76], as well as their technical and climatological publications, where this threshold is considered a standard for the beginning of the growing season under temperate continental climate conditions. After establishing the database of daily active temperatures, their cumulative values were calculated by successive summation from 1 January for each individual day until reaching the days of the year (DOY) corresponding to the occurrence of the phenophases: BBCH 60, BBCH 65, and BBCH 69. This procedure was conducted for each of the 20 research years, with separate analyses performed for Border Forsythia and Double Japanese Rose. The thermal requirements of the investigated taxa were precisely defined through a comparative analysis of phenological phases and climatic fluctuations [75].

### 4.3. Statistical Analysis

For a comprehensive analysis of the twenty-year phenological dataset (2007–2026), a statistical framework combining descriptive and inferential statistical methods was applied, with an emphasis on non-parametric tests [72]. The direction, statistical significance, and magnitude of monotonic trends in the time series (DOY and GDD) were assessed using the non-parametric Mann–Kendall (MK) test and Sen’s Slope Estimator [77]. The interdependence and strength of correlations between the analysed variables were evaluated using Spearman’s rank correlation test (ρ), in accordance with the standard classification [78].

The analysis of the phenological seasonality of flowering and the central tendency of phenophase occurrence was conducted using circular statistical methods [79]. Calendar dates (DOY) were transformed into angular coordinates, from which the mean angles (μ) were calculated.

Statistical data processing and trend analysis (Mann–Kendall, Sen’s slope, Spearman’s rank correlation, and non-linear regression) were performed using XLSTAT software (version 2022, Addinsoft, Paris, France). Circular statistical analyses were conducted using BioEstat software (version 5.3, Instituto Mamirauá, Belém, Pará, Brazil). The ArcGIS/ArcMap GIS environment (version 10.8, Esri, Redlands, CA, USA) was used for spatial analysis and data visualisation.

## 5. Conclusions

The 20-year study (2007–2026) addresses a methodological gap in urban ecophenology by introducing non-linear and circular statistical modelling, enabling a precise analytical distinction between regional climatic variability and local anthropogenic modifications associated with the urban heat island effect. The findings confirm that the internal dynamics of the climate system remain dominant at the macro scale, while accompanying microclimatic fluctuations are losing their historical stationarity and inducing specific, taxon-dependent phenodynamic responses in ornamental woody vegetation. Quantitative analysis identified two divergent ecophysiological mechanisms: a model of phenological compression in the hybrid *Forsythia × intermedia*, in which accelerated thermal forcing leads to a shortened flowering cycle and premature flower senescence, and a model of non-linear phenological expansion in the cultivar *Kerria japonica* ‘Pleniflora’, whose high phenological plasticity and successive bud opening extend flowering by more than one month, thereby enabling effective buffering of intra-seasonal stress. Circular statistical analysis confirmed high calendar synchronisation of the initial phenophases in both taxa under the influence of stable genetic sensitivity thresholds, while asynchrony and non-linear convergence in thermal requirements during the terminal stages demonstrate the distinction between calendar-based and thermal predictors in late spring. The synchronous shift in both taxa towards warmer temperature classes positions them as reliable bioindicators of local climate change, while the observed stability of their calendar patterns provides a robust empirical basis for the selection, introduction, and sustainable management of climate-resilient green infrastructure. Although the study is contextually linked to a specific urban core and does not incorporate socio-economic variables, its fundamental value lies in demonstrating the need to move beyond simplified linear approximations in biological forecasting. Future research will focus on validating the non-linear models in cities with different geomorphological characteristics and testing a broader range of plant taxa, with the ultimate aim of developing a dedicated GIS-based software tool using the ArcGIS/ArcMap software environment for the automated simulation of microclimatic changes and the development of a standardised index of urban climate resilience for vegetation.

## Figures and Tables

**Table 1:** Results of the Mann–Kendall (MK) and Sen’s slope tests for seasonal (winter and spring) temperature anomalies during the period 2007–2026 relative to the 1961–1990 reference period (*p* < 0.05). Legend: Tmean (mean air temperature).

Variable/Parameters	Kendall’s Tau	*p*-Value	Sen’s Slope
Tmean	Winter		
	0.284	0.086	0.136
	Spring		
	−0.042	0.820	−0.007

**Table 2:** Nonlinear regression mathematical models of phenological responses (DOY—day of year; BBCH—Biologische Bundesanstalt, Bundessortenamt und CHemische Industrie scale) as a function of accumulated growing degree days (GDDs) for the taxa *Forsythia* × *intermedia* and *Kerria japonica* ‘Pleniflora’ during thermally excessive seasons (“very warm” and “warm” years) in Belgrade (2007–2026).

Parameter	*Forsythia × intermedia*	*Kerria japonica* ‘Pleniflora’
“Very warm” years		
DOY BBCH 60	59.296 * exp (1.17548 × 10^−5^ * Year)	157,478 exp (−0.00379343 * Year)
DOY BBCH 65	39,435.9 exp (−0.00317361 * Year)	178,612 exp (−0.00382719 * Year)
DOY BBCH 69	1166.48 exp (−0.00124735 * Year)	0.000806246 exp (0.00595825 * Year)
GDD BBCH 60	9.1288 × 10^−5^ exp (0.0070617 * Year)	24.6036 exp (0.00107468 * Year)
GDD BBCH 65	0.00154497 exp (0.00574547 * Year)	2.91852 exp (0.00221352 * Year)
GDD BBCH 69	0.00210354 exp (0.00597589 * Year)	2.70665 × 10^−6^ exp (0.00966589 * Year)
“Warm” years		
DOY BBCH 60	358,963 exp (−0.0042596 * Year)	7833.26 exp (−0.00223686 * Year)
DOY BBCH 65	3.07569 × 10^6^ exp (−0.00528227 * Year)	7400.44 exp (−0.00218291 * Year)
DOY BBCH 69	295,926 exp (−0.00394385 * Year)	0.206206 exp (0.00323208 * Year)
GDD BBCH 60	3.17664 × 10^−5^ exp (0.00750437 * Year)	0.000984914 exp (0.00608059 * Year)
GDD BBCH 65	7417.63 exp (−0.00194792 * Year)	0.00103503 exp (0.00612712 * Year)
GDD BBCH 69	2.59715 × 10^6^ exp (−0.00442919 * Year)	0.00250029 exp (0.00625848 * Year)

**Table 3:** Results of the Rayleigh test for the assessment of the seasonality of thermal accumulation (GDD) and the directionality of flowering phases BBCH (Biologische Bundesanstalt, Bundessortenamt und CHemische Industriescale) 60, 65, 69 in *Forsythia × intermedia* (1) and *Kerria japonica* ‘Pleniflora’ (2), at a significance level of *p* < 0.05 (Belgrade, 2007–2026).

Taxon Parameter	(1)	(2)	(1)	(2)	(1)	(2)	(1)	(2)	(1)	(2)	(1)	(2)
	DOY BBCH						GDD BBCH					
	60		65		69		60		65		69	
Vector r	0.9894	0.9845	0.9867	0.9853	0.9887	0.9787	0.8425	0.7965	0.8509	0.84	0.7328	0.2475
Rayleigh R	19.788	19.689	19.733	19.706	19.774	19.575	16.850	15.929	17.019	16.801	14.655	4.9501
Rayleigh Z	19.578	19.384	19.470	19.415	19.550	19.159	14.197	12.687	14.481	14.114	10.738	1.2252
u	4.451	4.451	4.451	4.451	4.451	4.451	4.451	4.451	4.451	4.451	4.451	2.958
*p* value	<0.01	<0.01	<0.01	<0.01	<0.01	<0.01	<0.01	<0.01	<0.01	<0.01	<0.01	ns *
Mean angle	62.499	79.629	67.635	84.475	98.767	136.93	131.47	214.59	155.83	251.02	350.39	-

ns *—statistically non-significant; the calculated mean angle was considered unreliable and was therefore excluded.

**Table 4:** Results of the Watson–Williams test and circular correlation parameters for calendar days (DOY) and thermal accumulations (GDD) of flowering phenophases BBCH (Biologische Bundesanstalt, Bundessortenamt und CHemische Industriescale) 60, 65, 69 in *Forsythia* × *intermedia* (1) and *Kerria japonica* ‘Pleniflora’ (2) in Belgrade (2007–2026), at a significance level of *p* < 0.05.

Taxon Parameter	(1)	(2)	(1)	(2)	(1)	(2)	(1)	(2)	(1)	(2)	(1)	(2)
	DOY BBCH						GDD BBCH					
	60		65		69		60		65		69	
Watson–Williams test												
F	32.3156		29.0769		127.6302		48.5943		74.8509		12.0155	
*p* value	<0.0001		<0.0001		<0.0001		<0.0001		<0.0001		0.0017	
Circular correlation												
r_aa_ *	0.6309		0.5427		0.2384		0.4617		0.2715		−0.0299	
*p* value	0.0028		0.0134		0.3113		0.0675		0.2469		0.0094	

* correlation coefficient.

## Data Availability

The climatology data are freely available at https://www.hidmet.gov.rs/eng/meteorologija/klimatologija_produkti.php (accessed on 15 June 2026). The data from the Republic Hydrometeorological Service of Serbia, which cannot be published, was accessed multiple times.

## Supplementary Materials

The following supporting information can be downloaded at https://www.mdpi.com/article/10.3390/plants15172682/s1; Table S1: Trends in phenological patterns of primary flowering of *Forsythia* × *intermedia* and *Kerria japonica* ‘Pleniflora’ in Belgrade: Mann–Kendall results in conjunction with Sen’s slope tests for the twenty-year research period, (*p* < 0.05); Table S2: Correlation analysis (Spearman’s correlation coefficients—black color and associated *p*-values—red color) of the relationship between DOY and GDD values for *Forsythia × intermedia* and *Kerria japonica* ‘Pleniflora’ in the Belgrade area (2007–2026; *p* < 0.05).
