# Supplementary material for: Comparative Study of Thermal Accumulation and Adaptive Responses of Forsythia × intermedia Zabel and Kerria japonica (L.) DC. ‘Pleniflora’ Under Conditions of Climatic Fluctuations
Source: Plants (Basel). 2026 Aug 31;15(17):2682. doi: 10.3390/plants15172682 (PMC13567990; doi:10.3390/plants15172682)
Supplement: Supplementary file 1 [file plants-15-02682-s001.zip › plants-4523132-supplementary.pdf]

Table S1. Trends in phenological patterns of primary flowering of *Forsythia × intermedia* and *Kerria japonica* 'Pleniflora' in Belgrade: Mann-Kendall results in conjunction with Sen's slope tests for the twenty-year research period, ($p<0.05$).

Parametar\Test	Kendall's tau	p-value	Sen's slope	Kendall's tau	p-value	Sen's slope
Takson	*Forsythia × intermedia*			*Kerria japonica* 'Pleniflora'		
60BBCH DOY	-0.165	0.329	-0.544	-0.107	0.536	-0.303
65BBCH DOY	-0.277	0.097	-1.000	-0.086	0.625	-0.250
69BBCH DOY	-0.153	0.363	-0.388	0.377	**0.023***	1.243
60BBCH GDD	0.358	**0.030***	2.306	0.147	0.381	2.041
65BBCH GDD	0.063	0.721	0.678	0.179	0.284	2.072
69BBCH GDD	0.105	0.538	1.080	0.411	**0.012***	17.299

* Highlighted figures indicate statistically significant values

Table S2. Correlation analysis (Spearman's correlation coefficients - black color and associated p-values - red color) of the relationship between DOY and GDD values for *Forsythia × intermedia* and *Kerria japonica* 'Pleniflora' in the Belgrade area (2007–2026; $p<0.05$).

Variables	60BBCH	65BBCH	69BBCH	60BBCH	65BBCH	69BBCH
	DOY			GDD		
Forsythia × intermedia						
60BBCH DOY		**0.954***	**0.761***	**-0.558***	**-0.557***	0.058
65BBCH DOY	**<0.0001***		**0.701***	**-0.608***	**-0.464***	0.053
69BBCH DOY	**0.000***	**0.001***		**-0.448***	**-0.485***	0.190
60BBCH GDD	**0.012***	**0.005***	**0.049***		**0.744***	0.307
65BBCH GDD	**0.012***	**0.041***	**0.032***	**0.000***		0.293
69BBCH GDD	0.809*	0.826	0.420	0.188	0.209	
Kerria japonica 'Pleniflora'						
60BBCH DOY		**0.986***	0.006	0.173	0.170	-0.123
65BBCH DOY	**<0.0001***		0.085	0.097	0.115	-0.069
69BBCH DOY	0.982	0.982		-0.024	0.233	**0.923***
60BBCH GDD	0.463	0.463	0.921		**0.898***	0.072
65BBCH GDD	0.473	0.473	0.321	**<0.0001***		0.385
69BBCH GDD	0.605	0.605	**<0.0001***	0.762	0.095	

*Highlighted figures indicate statistically significant values
